# Improving the Properties of Laccase Through Heterologous Expression and Protein Engineering

**DOI:** 10.3390/microorganisms13061422

**Published:** 2025-06-18

**Authors:** Guoqiang Guan, Beidian Li, Ling Xu, Jingya Qian, Bin Zou, Shuhao Huo, Zhongyang Ding, Kai Cui, Feng Wang

**Affiliations:** 1 School of Food and Biological Engineering, Jiangsu University, Zhenjiang 212013, China; ggqyxq@ujs.edu.cn (G.G.); 2222318044@stmail.ujs.edu.cn (B.L.); qianjingya@ujs.edu.cn (J.Q.); binzou2009@ujs.edu.cn (B.Z.); huo@ujs.edu.cn (S.H.); 2 Key Laboratory of Carbohydrate Chemistry and Biotechnology, Ministry of Education, School of Biotechnology, Jiangnan University, Wuxi 214122, China; bioding@163.com; 3 Institute of Feed Research, Chinese Academy of Agricultural Sciences, Beijing 100081, China; cuikai@caas.cn

**Keywords:** laccase, heterologous expression, fungi, bacteria, enzyme engineering

## Abstract

Laccase, a member of the blue multicopper oxidase family, is widely distributed across diverse taxonomic groups, including fungi, bacteria, plants, and insects. This enzyme drives biocatalytic processes through the oxidation of phenolic compounds, aromatic amines, and lignin derivatives, underpinning its significant potential in the food industry, cosmetics, and environmental remediation. However, wild-type laccases face critical limitations, such as low catalytic efficiency, insufficient expression yields, and poor stability. To address these bottlenecks, this review systematically examines optimization strategies for heterologous laccase expression by fungal and bacterial systems. Additionally, we discuss protein engineering for laccase modification, with a focus on the structural basis and active-site redesign. The comprehensive analysis presented herein provides strategic suggestions for advancing laccase engineering, ultimately establishing a theoretical framework for developing high-efficiency, low-cost engineered variants for large-scale biomanufacturing and green chemistry applications.

## 1. Introduction

Laccase (phenylphenol: oxyredox enzyme, EC1.10.3.2) is a member of the blue polycopper oxidase family. The first laccase was discovered in the latex of the sumac *Rhus vernicifera* and subsequently tested in fungi, insects, and bacteria [1,2,3,4,5,6]. Structurally, laccase exists in the form of monomers, homotetramers, heterodimers, or polymeric glycoproteins. Laccase usually consists of four copper atoms organized into three type centers (types 1, 2, and 3). A paramagnetic type 1 copper (T1Cu) is responsible for its characteristic blue color; a type 2 copper (T2Cu) and two type 3 coppers (T3Cu) are distributed between different binding sites, forming a trinuclear cluster, and reduce oxygen to water [7,8]. This unique “four-copper” structure is maintained by a highly conserved internal electron-transfer pathway: one of the oxygen molecules is reduced to two water molecules [9,10]. The oxygen in the air is consumed without any toxic by-products, the only result being water. Therefore, laccase is also called a “green catalyst” [6,11,12,13].

Laccase can catalyze the oxidation of various substrates, such as phenols, anilines, polyphenols, and even some inorganic compounds [14,15,16,17]. It has been used in many industries, including environmental protection and industrial waste bioremediation, textile dyes, the paper and food industries, biological monitoring, and organic synthesis [17,18,19,20,21]. However, laccase applications are still limited by certain factors, such as the enzyme’s high cost, pH, thermo-instability, low expression rate, poor adaptability, and specificity [19,22,23,24,25]. Therefore, many studies have been conducted to alleviate these problems and improve laccase expression and performance.

Physical and chemical methods, including culture optimization, new strain isolation, co-culture, and chemical modification, are being examined to improve laccase expression rates [26,27,28,29,30,31,32,33,34,35,36]. Due to its structure, laccase is capable of different types of modifications. Fungi and yeast were isolated from delignified biomass in hilly areas of India, Haryana, and an airborne environment. Meanwhile, fungi–yeast interactions led to a 10-fold increase in secreted levels of a laccase isozyme (6532 U/mL) [37]. Three white-rot fungi—*Cerrena unicolor*, *Phlebia lindtneri*, and *Pycnoporus sanguineus*—were cultured under different light conditions: dark, white, red, blue, and green. The results showed that blue light effectively promoted laccase production in *Cerrena unicolor* and *Pycnoporus sanguineus*, whereas the activity of *Phlebia lindtneri* laccase reached 344.23 U/mL. A higher laccase yield (45.0%) was obtained with chemically modified acid anhydride [38]. Although physical and chemical methods can, to a certain extent, effectively improve laccase expression, there are still certain challenges, such as difficulties in studying gene function and low toxicity [39,40,41,42,43,44] and efficiency [38,45,46]. In addition to physical and chemical techniques, heterologous expression has also been employed as an efficient approach to modifying laccase [47,48,49,50,51,52]. There are many ways to modify laccase properties [49,52,53,54,55,56].

Given the current challenges of laccase modification, protein or genetic engineering [10,57,58,59,60,61,62,63,64,65,66] is the best approach to changing catalytic efficiency, selectivity [10,57], degradability [67,68], and organic solvent tolerance. There are many potential strategies for modifying laccase properties. The most widely used approaches include directed evolution [69,70], site-directed mutagenesis [57,71], a combination of culture optimization and protein engineering [10,72], and recombinant gene expression. Site-directed mutagenesis improved laccase specificity and efficiency [10,57]. In addition, directed evolution, laccase gene expression [73,74], and some combination strategies [75,76] have successfully generated ideal laccase characteristics [77]. Novel laccases were able to degrade substrates without a mediator [67]. Using these tools to engineer laccase is conducive to further understanding the structure–function relationship [78].

Modification at the molecular level might facilitate the utilization of laccase in bioengineering [53,79,80,81,82,83]. Protein engineering not only effectively improves the catalytic performance of the enzyme but also eliminates the need for repetitive and massive screening [84]. With directed evolution, specific problems might be encountered that require a specific analysis and a reasonable design in order to optimize laccase’s oxidation ability; this might include changing the redox potential between the laccase and the substrate or altering electron transfer. Modulating the substrate and the enzyme’s binding pocket adjusts the laccase redox capacity [85]. Protein or genetic engineering can effectively solve the problem of laccase stability under high demands and harsh environments in industrial applications [86].

Laccase has been extensively investigated in recent studies. Abhinashi Singh Sodhi et al. systematically outlined various strategies for enhancing its heterologous expression. The authors provided a comprehensive overview of laccase’s diverse sources, catalytic mechanisms, and production parameters, with a focus on its sustainable production via solid-state fermentation and methods for improving its quality through protein engineering and co-culture techniques. These advancements offer critical technical support for enzyme engineering and industrial biocatalysis [10]. The review by Milica Crnoglavac Popović et al. provides a detailed overview of recent advances in the heterologous expression of laccase (LAC), including key experimental findings, underlying principles, and challenges identified in these studies. The authors discuss yeast-based oxidoreductase expression systems, highlighting their advantages in post-translational modification and secretory pathways. Furthermore, their review explores emerging strategies for enhancing fermentation yields, such as directed evolution for improved enzyme thermostability, synergistic protein-strain engineering to optimize host compatibility, and advanced high-throughput screening methods, such as in vitro compartmentalization, flow cytometry, and microfluidic technologies. These approaches address critical bottlenecks in large-scale production while enabling the precise control of enzyme properties for industrial applications [87]. Yi et al. published a review of thermostable laccase and its current applications in lignin-first biorefineries. They focused on the sources of thermostable laccases, including those isolated from extreme environments or derived from reconstructed scenarios, as well as methods for their production, such as rational design and directed evolution [9]. Ilaria Stanzione et al.’s review [88] particularly highlighted the large-scale industrial application of engineered laccase. The main heterologous expression hosts for laccase production, namely fungi, bacteria, and yeast, were extensively reviewed by Zuzana Antos et al. [89] and Martinkov L. et al. [90]. In addition, Brandt Bertrand et al. discussed strategies for improving laccase, including traditional production strategies, genetic engineering, and chemical modification [91]. A number of studies have reviewed laccase applications in the production and development of some new technologies [92,93,94,95,96]. Among these are a few reviews on improving enzyme expression via allogenic expression and even fewer on protein engineering.

This article summarizes the progress to date in improving enzyme expression, which has mainly been achieved through heterologous expression and protein engineering. We review a variety of hosts suitable for laccase production, focusing on fungi and bacteria, which can be used to produce various industrial proteins. At the same time, this review summarizes the technical basis for modifying laccase through protein engineering, illustrating the relevant structural basis for laccase modification and active sites, which can affect its characteristics. Heterologous expression improves laccase expression levels and stability, overcoming the disadvantages of natural laccase to a certain extent. We provide information on how to change the structure of laccase at the molecular level through genetic engineering combined with protein engineering. Mutations can improve the catalytic efficiency of laccase, as well as its substrate affinity, stability, activity, and kinetic parameters. The content of this review can guide further developments in laccase modification.

## 2. Hosts for Heterogeneous Expression

### 2.1. Heterogeneous Expression with Bacteria as Hosts

The heterogeneous expression of laccase systems using bacteria as hosts continues to be developed; one such system that has been frequently reported is *E. coli*. Many sources of laccase have been used for its heterologous expression (Table 1). Isopropyl β-D-1-thiogalactopyranoside (IPTG) and copper ions (Cu^2+^) are widely utilized to induce laccase expression in *Escherichia coli*. IPTG, a lactose operon inducer, initiates T7 RNA polymerase-dependent transcription by binding to the lac repressor (*LacI*), enabling high-level recombinant protein production [97]. Because IPTG is structurally similar to lactose, it can induce protein expression in the same way. Therefore, it is possible to induce laccase gene expression using the T7 promoter [98]. IPTG can directly enter *E. coli* cells but is not consumed in bacterial metabolism. However, it is an expensive and potentially toxic chemical [99]. In contrast, Cu^2+^ acts through dual mechanisms: (1) it stabilizes the laccase catalytic center via coordination with histidine residues, and (2) it activates metal-responsive promoters (e.g., *copA*) to enhance transcription efficiency [100]. Laccase yields can be elevated 3.1-fold by the synergistic use of these inducers (e.g., 0.5 mM IPTG + 0.1 mM CuSO_4_) compared to single inducers. In addition, IPTG combined with copper ions was suitable for the expression of laccases from *Catenuloplanes japonicus* and *Streptomyces viridochromogenes* with *E. coli* as the host [48,101]. Elevated inducer concentrations not only suppress recombinant protein expression due to heightened metabolic burden [102] but also raise production costs. Conversely, suboptimal inducer levels fail to reach maximum yield thresholds [102,103]. Notably, the nonlinear correlation between inducer dosage and cellular viability necessitates precision modulation strategies—such as phased induction protocols or real-time feedback systems—to identify cost–yield equilibria while maintaining host fitness. Meanwhile, the auto-induction strategy was more suitable for laccase expression from *Bacillus Vallismortis fmb-103* in *E. coli* [98]. Optimizing laccase expression conditions is essential for obtaining high yields [99]; selecting these conditions is therefore the most critical step in improving laccase expression.

#### 2.1.1. Cloning and Heterologous Expression

With the rapid development of DNA sequencing technology and biological information, the gene sequence for laccase biosynthesis in the microbial genome was found. Genetic design is becoming increasingly important, and primer design and vector construction are essential in the cloning process.

Laccase genes are usually derived from one of two sources. The laccase gene from the original host can be heterologously expressed in *E. coli* [117], or it can be synthesized by selecting a protein encoded by a homologous gene and optimized using gene software and a laccase template. Yang et al. screened a putative laccase gene, LacSM, from *Sordaria macrospora* k-hell via genome mining. Subsequently, the gene was cloned and highly expressed in *Escherichia coli* [105]. There are also two sources of primers: those generated by other researchers [106] and those synthesized based on a database [22,103,107,109]. Primers were constructed on the basis of UniProt Data Analysis [107], and others were based on a laccase gene open reading frame [22]. In addition, primers were designed by deleting the signal peptide coding sequence [118]. The laccase gene sequence is the foundation for primer design. The expression plasmid POX1Ab/pET-22b (+) was introduced into Escherichia coli DH5α competent cells via heat shock (42 °C, 45 s) to enable recombinant laccase production [108].

#### 2.1.2. Enzyme Properties from Bacterial Hosts

Recombinant expression may produce enzymes with varying molecular weights. Currently, most bacterial recombinant laccases are from *E. coli*. The molecular weight of recombinant laccase is about 20–80 KDa, and those of thermostable recombinant laccases from *Geobacillus* sp. *JS12* [22] and *Geobacillus* sp. strain WSUCF1 [104], expressed in *Escherichia coli*, are 30 kDa and ~30 kDa, respectively. The *Thermus thermophilus* HJ6 recombinant laccase had a signal band of ~27 kDa in SDS/PAGE [119]. Laccases with low molecular weights of 24 kDa and 43 kDa from *Pseudomonas* spp. [107] and *Yersinia enterocolitica* strain 8081 [106] were also reported. Due to their small size, recombinant laccases have many important industrial applications, such as lignin degradation, the hydrolysis of lignocellulosic biomass, and biocatalytics. Native and recombinant *Trametes sanguineus* laccases have shown similar bands at ~67 kDa [120]. The molecular weights of the native proteins are 88 and 90 kDa in SDS-PAGE. However, the molecular weight of SvSL laccase after boiling is 39 kDa [101]. The 200 kDa band for the native laccase from *Catenuloplanes japonicas* and the 37 kDa band for CjSL [48] indicated that recombinant expression can greatly change the enzyme’s molecular weight. This is because, during the heterologous expression process, plasmids produce soluble protein-like substances in the cytoplasmic components. These substances migrate to positions above the upper limit of the low-molecular-weight markers in the SDS–polyacrylamide gel and exhibit DMP oxidation activity, altering the enzyme’s molecular weight [121].

Recombinant laccase expressed in *E. coli* has garnered strong interest in the last few decades because it has significant characteristics that improve its tolerance to harsh environments, such as higher temperatures and wider pH ranges [22,122]. Several authors have shown that the temperature and pH characteristics of laccase after recombination are significantly improved. This could be explained by changes in laccase after its expression, resulting in improved temperature and pH ranges. The optimal reaction temperatures for LacSM to oxidize ABTS, SGZ, 2, 6-DMP, and guaiacol are 50, 55, and 60 °C, respectively [105]. The recombinant protein has different optimal pH values with different substrate reaction systems; for example, the optimal pH values of guaiacol and 2, 6-DMP oxidation were 6.0 and 6.5 [22]. Recombinant laccases have wider temperature and pH ranges compared to their native counterparts [107]. The laccase gene LacHazai from the deep-sea halophilic bacterium Halomonas alkaliantarctica was cloned and heterologously expressed in Escherichia coli, where the enzyme demonstrated a broad pH activity range of 5.0–9.0 and thermal stability between 25 and 65 °C [111]. However, laccase generally showed the highest enzyme activity at pH 3–7 and temperatures of 30–70 °C. In addition, the appropriate temperature not only maximizes the expression of laccase during incubation but also reduces enzyme activity retention [106,108].

Much research has focused on the significant influence of metal ions on enzyme and protein activity. FeSO_4_ and CuSO_4_ can improve laccase activity [104]. However, Fe^2+^ plays a positive regulatory role in inhibiting enzyme activity [22,105]. The recombinant laccase Lac3833 was stable in buffer solutions containing Na^+^, K^+^, or Mg^2+^ (1–100 mM), retaining over 80% enzymatic activity. While it maintained 60% residual activity in Ca^2+^ and Mn^2+^ solutions, Fe^2+^/Fe³^+^ exerted significant inhibitory effects (activity < 20%). Notably, Lac3833 preserved > 70% activity at three Cu^2+^ concentrations (0.1–10 mM), indicating robust catalytic performance under common metal ion conditions [115]. One study examining laccase restructuring used the productive, active part of Fe^2+^, which had different effects on different recombinant laccases [112].

#### 2.1.3. Advantages and Disadvantages of Laccase Expression in Bacteria

Inclusion bodies are formed in the process of heterologous expression in bacterial hosts; their solubilization is integral to heterologous expression, and methods in this regard have been reported [22,106]. TtSLAC and lacG from *Thermus thermophilus* HJ6 and *Geobacillus* sp. JS12 were successfully overexpressed in *E. coli* cells; however, the majority of laccases were found in inclusion bodies [22,119].

Bacterial laccases exhibit high substrate specificity and also outnumber their fungal counterparts. They have a wider optimal pH range, greater tolerance to temperature, and low sensitivity to alkaline environments [111,119]. The laccase yield was higher in *Escherichia coli* than in a fungal host. The co-expression of laccase from *Phomopsis* sp. XP-8 (CCTCCM209291) in *E.coli* with a small heat shock protein (HSP20) could increase the enzyme’s activity and heat stability [123]. The two-domain laccase from *Cateuloplanes japonicus* expressed in *E. coli* demonstrated an increased range of oxidized phenolic substrates and greater thermal stability [48]. Bian et al. cloned the laccase gene from *Bacillus vallismortis* FMB-103, codon-optimized it for heterologous expression in Escherichia coli, and further enhanced extracellular production through methanol induction in the extracellular medium, achieving a 1.8-fold increase in laccase activity compared to non-induced controls [114]. Optimizing the conditions for recombinant laccase expression in *E.coli* resulted in high extracellular production of LacA; based on this, a chimeric enzyme was designed to improve laccase activity [53,99,115,124].

It is worth noting that, although bacterial laccases can be highly stable under harsh conditions, their activities and production yields are usually very low. Methanol is provided as the inducer and carbon source in order to promote protein production during the induction phase. The target gene cloned from *Bacillus vallismortis* FMB-103 was codon-optimized for heterologous expression in *Escherichia coli*. Methanol induction (6%, *v*/*v*) was applied extracellularly, resulting in a maximum enzymatic activity yield of 1545.6 U/L [114]. The excess methanol in the medium can, however, have an adverse effect on cell growth and production yield [75].

However, bacterial systems for heterologous expression face inherent limitations, particularly in their inability to perform eukaryotic post-translational modifications (PTMs), such as glycosylation or disulfide bond formation, which are often essential for proper protein folding and functional activation [125]. Additionally, while certain recombinant laccases exhibit thermotolerance under standard assay conditions, their thermal stability is time-dependent, with rapid activity decay observed beyond 60 °C over prolonged incubation [126].

### 2.2. Heterologous Expression in Fungi as Hosts

In their capacity as hosts for heterologous expression, fungi are mainly divided into two groups: yeast and filamentous fungi. For heterologous expression in yeast, *Pichia pastoris* [87,127,128] is the main host, though *Saccharomyces cerevisiae* [87,129,130] and *Yarrowia lipolytica yeast* [131] have also been studied. In addition, laccase has been successfully expressed in *Trichoderma* [132]. Based on a summary of published laccase sources and expression hosts (Table 2), we concluded that fungal hosts for heterologous expression are essential in bioengineering. The main inducers in fungi are Cu^2+^, lactose, and methanol. Flávia F. Magalhães et al. enhanced laccase production in Pichia pastoris through a combined strategy of daily methanol induction (1% *v*/*v*), copper ion supplementation (0.1 mM CuSO_4_), and temperature-controlled incubation at 25 °C [39].

Among current filamentous fungal heterologous expression systems, the predominant hosts include *Aspergillus nidulans*, *Trichoderma atroviride*, and *Trichoderma reesei*, which have been utilized to produce laccases derived from thermophilic bacteria and basidiomycetes, such as *Pycnoporus sanguineus* (syn. *Trametes sanguinea*), with enzymatic characterization performed using standardized ligninolytic substrates, e.g., 2,2′-azino-bis(3-ethylbenzothiazoline-6-sulfonic acid) (ABTS), guaiacol, syringaldazine, and o-dianisidine.

#### 2.2.1. Cloning and Heterologous Expression in Fungi

The codon-optimized laccase gene sequence was obtained from GenBank and synthesized using the ABI 3900 High-Throughput DNA Synthesizer; this was followed by directional cloning into expression vectors to construct recombinant plasmids [132] for expression in each host. Meanwhile, the combination of promoter, signal peptide, and host is important for obtaining the best protein expression [147]. Liu et al. screened vanillin-sensitive laccase promoters using green fluorescent protein (GFP) as a reporter; they identified two endogenous vanillin-responsive promoters and established a novel strategy for enhancing heterologous laccase expression in *Pichia pastoris* [145]. In *Pichia pastoris*, the alcohol oxidase (AOX1) promoter demonstrated superior induction efficiency for heterologous expression [133,148]. Critical studies revealed that hybrid promoter systems (e.g., Ppki-based constructs in *T. atroviride*) enhanced laccase expression by 47% through optimized transcriptional regulation [120]. Native *Pycnoporus sanguineus* promoters exhibited 72% sequence homology with *Aspergillus nidulans* promoter consensus regions, enabling efficient expression without synthetic modifications [140]. For plasmid transformation, the lithium acetate method achieved 1.2 × 10^3^ CFU/μg DNA efficiency in *Saccharomyces cerevisiae* [130], whereas electroporation protocols optimized at 1.5 kV generated 1.8-fold higher transformation rates in *P. pastoris* compared to chemical methods [118,135,149]. Notably, *Agrobacterium*-mediated transformation demonstrated 86% single-copy insertion efficiency in filamentous fungal hosts, which is particularly effective for *T. reesei* chromosomal integration [132]. Standardized vector systems, including pPIC9K (with α-factor secretion signal) [118], pJRoC30 (GAL1-regulated) [24], and PCB-lac (Pcbh1-driven) [132], were systematically validated in different host systems, with signal peptide–promoter combinations showing synergistic effects on protein secretion [47].

#### 2.2.2. Enzyme Properties from Fungal Hosts

The molecular weights of enzymes are typically confirmed by SDS PAGE. Typical recombinant laccase enzymes have molecular weights ranging from 50 to 110 kDa. The molecular weights of laccase genes from *Volvariella volvacea*, *Aspergillus* sp., and *Phlebia brevispora* were 54, 65, 69, and 110, respectively [127,134,150]. *Phlebia brevispora* BAFC 633 mainly produces a laccase of 60 kDa. However, the recombinant laccase had a signal band of 110 kDa in SDS PAGE, which is twice the predicted value [127]. The molecular mass of the purified recombinant laccase was consistent with its native or theoretical molecular weight [150]. Its molecular weight could also be affected by structural changes after N-glycosylation. After *N*-linked carbohydrates were removed from rLac2 and rLac3, the molecular weight changed from ~95 to 60 kDa [136].

Recombinant laccase has better pH activity profiles and enzyme thermotolerance compared to its natural counterpart [139,144]. The optimal pH and temperature ranges in fungal hosts are from 2 to 7 and from 20 to 70 °C, respectively. The recombinant laccase exhibited enhanced structural stability under alkaline conditions [144] but not in the acidic range. In recent years, studies have been performed on filamentous fungi. The optimal pH and temperature for the host *T. reesei* are reported to be 4 and 65 °C, respectively [132], showing excellent thermal stability. The recombinant laccase produced by *Pichia pastoris* is stable at 55 °C with ABTS as substrate [151]. In addition, the optimal temperature for the recombination of laccase from *Pycnoporus sanguineus* in *T. reesei* was 60 °C with the same substrate [132]. In studies with metal ions, Cu^2+^ greatly improved the laccase yield [152]. Metal ion screening revealed distinct regulatory effects on rFoLacc5 activity: Fe³^+^ completely inhibited enzymatic activity, while Cu^2+^ significantly enhanced catalytic performance. Divalent cations, including Mn^2+^, Co^2+^, Mg^2+^, Ni^2+^, K^+^, and Zn^2+^, exhibited partial inhibition (40–65% residual activity). Notably, Ca^2+^ displayed no significant impact on enzyme function [141].

#### 2.2.3. Advantages and Disadvantages of Laccase Expression in Fungi

Fungi have some advantages in heterologous expression. Yeast species serve as versatile microbial hosts with distinct advantages, including rapid growth kinetics, facile genetic manipulation, efficient protein secretion, eukaryotic post-translational modification capabilities, scalability in industrial fermentation processes, high biomass yields, and non-pathogenic GRAS (Generally Recognized as Safe) status [153,154]. *Pichia pastoris*, a methylotrophic yeast, utilizes methanol as its sole carbon source. This expression system enables straightforward genetic manipulation, achieves high-cell-density cultivation, and secretes recombinant proteins into the culture medium, simplifying purification. Eukaryotic post-translational modifications and stable genetic constructs further enhance its utility for recombinant protein production [155]. For instance, *T. reesei* has an excellent ability to secrete proteins extracellularly, which is helpful for downstream processes such as protein leaching and purification [132]. Given its high protein production and versatility in undertaking complex post-translational modifications, *Pichia pastoris* is now regarded as a successful protein production platform [135]. As heterologous expression hosts, fungi have the following advantages: (1) fungi produce extracellular laccase without lysis [9]; (2) yeast signal peptides constitute one of the strategies for enhancing recombinant laccase secretion; and (3) yeast reach a high cell density in fermentation. However, certain questions in this regard are worth pondering. Heterologous expression is an effective way to increase the production of the enzyme, but the degradation of foreign proteins by the host cannot be neglected [132]. A new laccase from *Agrocybe pediades* was expressed in *Saccharomyces cerevisiae* and enhanced the yeast’s yield and tolerance to inhibitors [139]. The functional expression of *Fusarium* sp. laccase in *Saccharomyces cerevisiae* was investigated, and culture condition optimization combined with protein engineering resulted in approximately 30-fold increased activity in the culture supernatant [72]. Therefore, choosing an applicable foreign protein and host is important for effective heterologous expression.

## 3. Comparison of Laccase Expression in Bacterial and Fungal

The hosts used for the heterologous expression of laccase are often fungi and bacteria. Each has unique advantages, and they can both increase laccase yield and stability to some extent. To improve laccase expression efficiency, gene-engineering strategies and induction/expression optimization are commonly used. However, due to their differences as hosts, bacteria and fungi warrant comparison.

We compared the differences in the expression process between bacterial and fungal hosts (Table 3). The source of recombinant bacterial laccase can be intracellular or extracellular, with most recombinant fungal laccases being extracellular. For the same culture duration, recombinant fungal laccase shows a higher yield than bacterial laccase. Fungal laccase production is commonly influenced by carbon and nitrogen sources, inducers, environmental conditions, etc. Bacterial laccase production is also affected by diverse factors, including pH, temperature, and nutrients. In contrast to fungal laccase (acid-stable), bacterial laccase is alkali-stable. Bacterial laccases are more active in alkali conditions and more thermostable than recombinant fungal laccases, as many of them can be activated at 80–90 °C. Bacterial laccases have a lower sensitivity and dependence on inhibitors and metal ions, are more amenable to heterologous expression, and are more easily modified via protein engineering.

## 4. Modification of Catalytic Properties of Laccase

### 4.1. Laccase Modification Design

In recent years, due to the increasing application requirements of laccase biotechnology, the structural modification of laccases has become a research hotspot in an effort to improve their characteristics. Based on the known laccase gene sequence and structural information, specific residue sites near the T1 copper center of the laccase from *Trametes* sp. C30 that interact through H-bonding and hydrophobic interactions (e.g., His481 and Asn288; His481; Asn288 and Asp230; and His481 and Asn288) [156], or the substrate-binding loops of *Thermus thermophilus* SG0.5JP17-16 laccase (e.g., D357 and K430 in loops 5 and 7) [157] are selected for mutation, and laccase gene engineering is carried out at the molecular level. This is achieved using site-directed mutagenesis [10,156], site saturation mutagenesis [72], directed evolution combined with rational or semi-rational methods [158], and optimized experimental strategies [75]. Site-directed mutagenesis or site saturation mutagenesis has become a promising approach to laccase engineering.

#### 4.1.1. Laccase Structure

##### Structure of Laccase Protein

**Figure 1:**
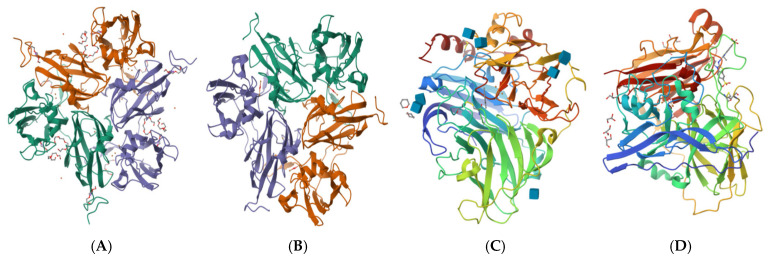
The three-dimensional structures of typical laccases (**A**), *Streptomyces coelicolor*; (**B**), *Streptomyces carpinensis*; (**C**), *Trametes versicolor*; (**D**), *Bacillus subtilis*. Structural data were obtained from the PDB database).

Specific structures are the basis of biological function. A protein’s functional structure determines its biological function. Schematic diagrams of the three-dimensional structures of typical laccases are shown in Figure 1. In laccase protein engineering, structural analysis mainly includes the laccase crystal structure and protein secondary structure. There are many laccase sources, and they all produce enzymes with different structures. For instance, Streptomyces coelicolor small laccase (SLAC) exhibits a trimeric architecture with a shallower substrate channel compared to its fungal counterparts [159]. The relationship between structure and functionality has been investigated in various structural analyses of laccases with different characteristics [160]. Such studies can be used to modify laccases at the molecular level. A series of mutants based on the known active sites for the substrate in the crystal structure of *Trametes versicolor* laccase showed improved properties [161]. The results of crystallographic experiments on small four-copper laccases showed that they contain only two domains [162]. This was the crystal structure of the first plant laccase. The crystal structure of corn laccase (*ZmLac3*) presents a compact and deep substrate-binding pocket; here, polarity and hydrophobicity are two of the key factors affecting catalysis [163]. The 7D5 laccase was developed in *Saccharomyces cerevisiae* and overproduced in *Aspergillus niger*. The obtained crystal structure was compared with that of wild-type basidiomycete PM1. The 7D5 laccase is heavier with more heterogeneous glycosylation, which creates a more oblate geometric structure [164]. With its complex topology and tight packaging, this type of domain is difficult to expand [59]. After adding mutations on the surface of the protein, it was found that the rigidification of certain loops would be affected, which has a beneficial effect on protein folding [164,165]. In addition, follow-up studies have been carried out on the ABTS-bound crystal structure of CotA from *B. subtilis* on the basis of that of laccase [57]. Crystallography and substrate docking research can aid in better understanding laccase’s structure–function relationship [72,166].

A comparative analysis of three laccase structures (blue, white, and yellow laccases) [167] concluded that different laccases would have different protein secondary structures, which may be one of the reasons for their divergent catalytic efficiencies and substrate specificities [86]. An X-ray diffraction analysis of the three laccase proteins showed that they all contain α-helix (10°) and β-sheet (22°) structures, but the intensity of these structures differed among the three laccases. The α-helix strength is greater in the white and yellow laccases than in the blue laccase, whereas the reverse is true of β-sheet strength. This structural divergence correlates with thermostability, as β-sheet-rich blue laccases exhibit higher thermal tolerance compared to their α-helix-dominant fungal counterparts [86,168]. In addition, Fourier transform infrared spectroscopy was used for protein analysis, enabling the identification of α-helices (1650–1658 cm^−1^), β-sheets (1620–1640 cm^−1^), amide I (1700–1600 cm^−1^), amide II (bands at under 1400 cm^−1^), and amides A and B (bands above 3000 cm^−1^). Some researchers used the known crystallographic structure of Lcc4 as a template to simulate a three-dimensional structure of *Botrytis cinerea* nLcc9; they obtained the structural composition of b-strands, α-helices, and 3/10-helices and then inferred the residues in the laccase domain [169]. The β-sheet content was reduced by 18% compared to wild-type laccase, likely due to loop rigidification [86], including domain 1 (D1, residues 22–149), domain 2 (D2, residues 167–306), and domain 3 (D3, residues 347–517). The circular dichroism spectrum of the protein can be used to estimate the secondary structure of the protein and determine whether it is correctly folded [170]. The recombinant laccase is mainly composed of 34.5% α-helices, 11.0% β-sheets, 23.1% β-turns, and 31.3% random coils [171]; the proportions of α-helices and β-sheets in the secondary structure differ between recombinant and wild-type laccases [165]. A comparison revealed that the secondary protein structure of the variant is not significantly different from that of the wild type. The Glu188Tyr mutation increased tertiary structural compactness via enhanced hydrophobic packing, improving thermostability 32-fold [84,86]. However, the increased compactness of the tertiary structure of the Glu188Tyr variant may be one of the reasons for the improved stability of the mutant [158]. In addition, laccase stability factors have also been discussed. Hydrophobic effects on proteins [156,166], the formation or breaking of hydrogen bonds [72,75], and salt bridges [59,172] have been found to be important factors affecting laccase properties.

Studying the structure of laccase is necessary to further exploring its biological function. This will help researchers develop various methods for effective functional and structural changes, improving laccase properties and utilization.

##### Copper Structure in Laccase

**Figure 2:**
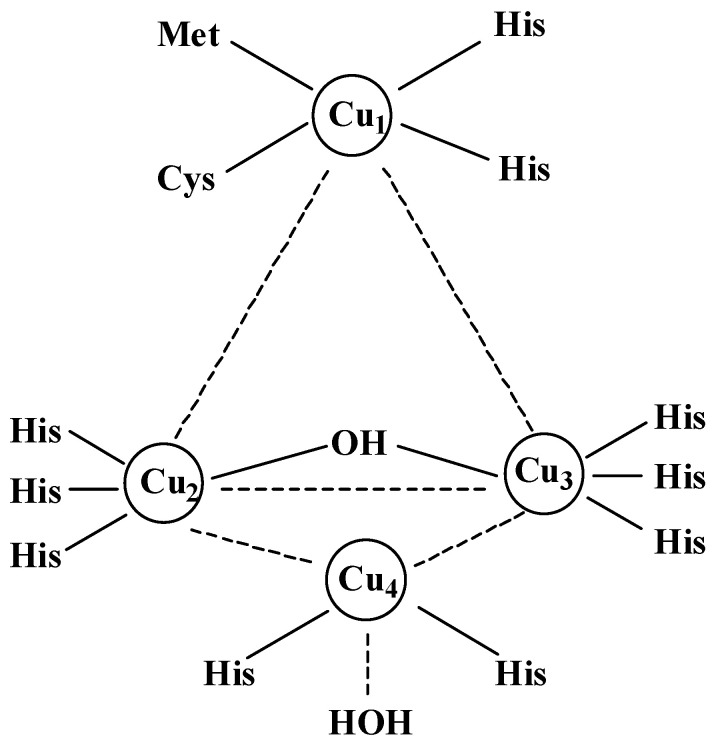
A schematic diagram of the CotA-laccase active site [184].

Laccase consists of three relatively stable domains, which combine to form an active structure. A typical diagram of the active site from CotA-laccase is shown in Figure 2. In the molecular structure of laccase, four copper ions are distributed in three structural domains, which are then divided into three types of copper sites according to their magnetic and spectral properties [173,174]. Type 1 copper (T1 Cu) is located in domain 3, which is where the substrate contacts laccase and provides electrons to type 1 copper. Type 2 copper (T2 Cu) is located in domain 1, and domain 3 has two copper ions. Domains 2 and 3 form a trinuclear T2/T3 center (TNC), where oxygen is reduced to water [175,176,177]. T1 copper is coordinated by two histidines, a cysteine, and an axial ligand; T2 copper is coordinated by two histidines and a water molecule; and the two copper atoms in T3 copper are each coordinated by three histidine residues [178]. Studies have also shown that the insertion of new copper ions in the T1, T2, and T3 sites of laccase affects its oxidation activity. A new Cu site (T4) is necessary for effective oxidase activity near the T1 site [179]. In the process of laccase catalysis, the substrate is oxidized near the T1 copper, and electrons are transferred to TNC through the Cys-His pathway [180]. The substitution of the axis residues at the T1 copper site in *Thermus thermophilus* SG0.5JP17-16 laccase decreased or increased the catalytic efficiency of laccase on guaiacol (**k**_cat_/*K*_m_) [178]. The hydrophobicity of the T1 copper axis residues could change the T1 copper environment and affect the catalytic activity of laccase [166]. At the same time, the increased hydrophobicity of the T1 copper axial residues increases the reduction potential [181]. The three domains contain multiple active sites for the substrate–laccase reaction. Among them, the T1-copper-type active site is commonly found in various laccase structures. These active sites are key factors in determining laccase’s catalytic ability [166,182], while substituting the residues in the substrate-binding pocket has an impact on its specificity and catalytic efficiency [57]. Some amino acid residues near the substrate-binding pocket of *Bacillus pumilus* W3 CotA-laccase [173] affect its catalytic ability. Furthermore, its methionine-rich helix and relative regulatory loop have an important influence on its activity [183]. The movement of the R-loop is associated with changes in the methionine-rich region, promoting the T1 copper site’s binding to the substrate. Meanwhile, the absence of domain 3 in the T1 copper center will affect enzyme activity [67]. In addition, studies have shown that domains 1 and 2 may have other mechanisms that affect laccase properties [67]. Understanding this structural analysis of laccase can help in its effective modification.

##### Laccase Gene and Function

Certain genes have a decisive effect on the pH, temperature, or substrate specificity of laccase. Nucleotide sequence changes can improve the catalytic efficiency of laccase on syringaldazine [181], as exemplified by the Y230R mutation in Streptomyces coelicolor laccase, which increased ABTS oxidation activity by 104% while broadening the enzyme’s range of pH tolerance to 3–9 through hydrogen bond network restructuring [185]. Replacing the bases in the selected target gene and performing site-directed mutations on specific bases of a known gene can change the corresponding amino acid sequence and protein structure. For instance, using domain-swap mutagenesis to replace domain 2 (D2, residues 167–306) generated the chimeric Lac-Vader variant with 3.4-fold higher activity and good thermal tolerance [86]. Changing the base sequence corresponding to the specific structure introduced a mutation in the rhlacc gene. Compared with the wild-type laccase, the mutant has a different optimal pH and substrate affinity [67]. The protein is truncated, resulting in the deletion of domain 3 containing the T1 copper center. The corresponding structural changes in laccase will affect its biochemical properties, as observed in Bacillus licheniformis laccase, where N-terminal truncation enhanced its solvent tolerance 3-fold in 20% DMSO [186]. The laccase LAC1 gene has 1821 bp encoding a protein composed of 570 amino acids. The protein base sequence contains seven N-glycosylation sites [187], and the degree of glycosylation can affect the enzyme’s characteristics.

#### 4.1.2. Design Method

**Figure 3:**
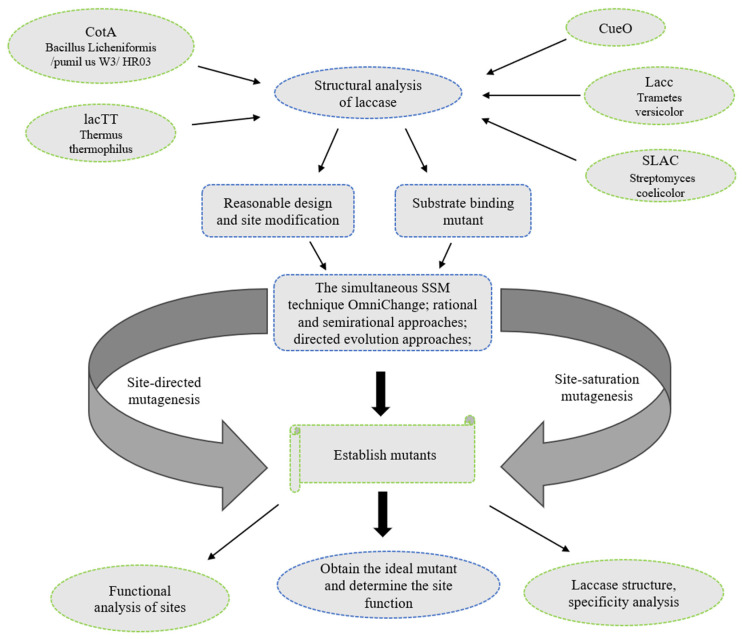
A schematic diagram of methods for engineered laccase.

Many potential techniques for engineering laccases have been presented in different studies; the most prominent of these are described below (Figure 3).

In site-directed mutagenesis, primers are designed based on existing genes and synthesized; then, templates are prepared, the obtained PCR products are processed, and positive clones are screened. Site-directed mutation and molecular simulation analysis are used to engineer laccase to increase its temperature stability, pH adaptation range, or substrate specificity. Site-directed mutagenesis modifications have focused primarily on the active site and substrate-binding pocket [84,182,188]. PCR primers can be selected by referring to the known laccase cDNA sequence [189]. First, a model or sequence comparison analysis is commonly carried out in the library, and then a series of mutants are obtained by using kits or PCRs for specific site modification. Isabel Pardo et al. [172] performed domain swapping and site-directed mutagenesis; first, they analyzed the crystal structure of the laccase protein, established a 3D protein structural model, and used the software Modeller in the UCSF Chimera graphical interface. The laccase crystal structure was established using Basidiomycetes PM1 and P. as templates to produce the lowest Z-score; then, the copper-domain-encoding gene in the genome was mutated to improve laccase stability. Site-directed mutagenesis studies have been carried out using the small two-domain laccase from *Streptomyces sviceus* [181]. After establishing a series of mutants, the base sequence of each is related to the characteristics of laccase; these sequences also affect its stability.

Researchers often design specific substrate-binding mutants based on their structural characteristics. Laccase may bind to the substrate in a low-energy configuration and high-binding model, which are most likely to be optimal. Homology modeling of laccase LAC3 was performed, followed by molecular docking experiments to predict the molecular-level binding affinity of the protein. Finally, the key amino acids were determined, and site-directed mutagenesis was used to introduce site mutations [156].

Wang et al. [75] selected a template for site-directed mutagenesis and used a fast mutagenesis system to construct mutants using PCR. Site-directed mutagenesis combined with codon optimization was used to clone and express mutant proteins in *Pichia pastoris*. Single-point and double-site mutations were achieved after two rounds of laboratory evolution [190]. Hui Ma et al. [173] engineered laccase via semi-rational methods of molecular docking and binding site saturation mutagenesis and obtained promising results. Mateljak and Alcalde [86] employed SCHEMA-RASPP recombination to design chimeric laccase variants, targeting conserved domains for modular swaps. Through rational consensus mutagenesis, combined with codon optimization using P. pastoris-biased codons, the evolved Lac-Vader mutant was heterologously expressed in Pichia pastoris, where it achieved a 9-fold improvement in thermostability and 32% higher catalytic ABTS efficiency compared to the parental OB-1 laccase. The laccase gene was cloned and expressed, and the crystal structure of Bacillus subtilis CotA laccase revealed that Trp94-mediated π-π stacking is critical for substrate recognition, enabling mutation modification experiments targeting electron-transfer pathways [57,166,183]. After designing site-directed mutagenesis primers for laccase, clone expression analysis was performed on PCR products [178,181] to obtain the desired characteristics. Preliminary computational research through molecular docking has also been carried out, and rational designs for mutation sites have been proposed after analyses of the internal structure of laccase [72,156]. On the other hand, there are also studies in which variant sites were designed through site-directed mutagenesis to determine how specific sites in the laccase structure, such as glycosylation [169] and N- and C-terminal sites [189], function. After designing, modeling, and analyzing the structure of laccase, molecular dynamics simulations were performed with precise single-residue mutations to obtain ideal mutants [59]. Researchers opt for a computational protocol, sequence comparison, or structural research [59,78]. On the basis of the simulation results, calculating the beneficial mutation sites and possible results of design analysis can help reduce laboratory time and greatly reduce screening [191,192].

Directed evolution processes the target gene directly at the molecular level; then, high-throughput screening methods are used to improve the performance of the target laccase. In recent years, there have been two main directed evolution techniques for obtaining improved laccases, which are based on the protein sequence and structure [191]. Through directed evolution and structural-analysis-based mutagenesis technology, each laccase is engineered to obtain improved properties or new functions. A directed laccase evolution experiment was successfully carried out in order to improve pH stability [193], enhance catalytic activity on specific substrates [194], and produce resistant laccase [195].

Some researchers have also regulated protease activity through polymer coupling [196]. Meanwhile, chemical mutagenesis has been used to manipulate the genome to increase laccase activity [197]. Modification and aggregation may have an effect on laccase activity [196].

### 4.2. Laccase Modification

#### 4.2.1. Protein Site Modification

##### Active Site

Crystal structure and nucleotide sequence analyses of laccase have elucidated the active sites in the three domains. Three conserved domains form the catalytic core: the T1 copper center (coordinated by two histidines, a cysteine, and an axial ligand), the T2/T3 trinuclear cluster, and substrate-binding loops [86]. These active sites determine the oxidizing ability of laccase on each substrate. At present, much research is being conducted on the modification of the T1 copper site in laccase structures. The most active sites are located at or near the center of T1 copper, and these are the most frequently targeted in laccase modifications, followed by amino acid residue replacement and axial ligand substitution [198]. Mutations at sites near T1 copper can increase or reduce laccase stability and catalytic efficiency. Based on molecular docking analysis and homology modeling, Hui Ma et al. [173] found specific substrate-binding sites near the T1 copper center in domain 3, including amino acids from 434 to 454; the Gln442 residue at the entrance to the substrate-binding pocket and its side chain could form a hydrogen bond with the carbonyl group of Thr438. The results showed that mutating the hydrophilic amino acid Gln442 to the hydrophobic amino acid Ala442 can effectively improve laccase substrate affinity. Residue 442 mainly determines substrate affinity, while residue 418 seems to significantly affect the conversion rate. Liu et al. [185] conducted molecular dynamics simulations and homology modeling of the *Streptomyces coelicolor* small laccase (SLAC), identifying critical substrate-binding residues (Tyr229/Tyr230) adjacent to the T1 copper center in domain 2. Structural analysis revealed that Tyr230 forms hydrogen bonds with Glu228 and His293 (bond lengths: 2.6–3.0 Å), while its hydrophobic interactions with Val290 and Met296 define the substrate channel geometry. Site saturation mutagenesis at position Tyr230 generated the Y230R variant, which increased ABTS oxidation activity by 104% and broadened the range of pH tolerance to 3–9 due to enhanced hydrogen bond networks and reduced steric hindrance. Concurrently, mutant molecular docking suggested improved substrate orientation (ΔG = −8.2 kcal/mol vs. −6.5 kcal/mol for wild type), with residue 230 primarily governing substrate affinity [185]. Hanqian Wang et al. [183] proposed a mechanism for the mutant to improve laccase activity. The methionine-rich helix and the relative regulatory loop (R-loop) of the mutant G304K underwent major conformational changes, which exposed the T1 copper site and improved the binding of the substrate and laccase compared with the wild-type CueO. Based on *Thermus thermophilus* SG0.5 JP17-16 (lacTT), experiments were designed to analyze four residues integral in laccase activity. Site-directed mutagenesis was used to study the four residues, E356, E456, D106, and D423, in lacTT catalysis. The results showed that the four residues are responsible for the stable structure of laccase’s active copper site. E356 is located at the substrate-binding site. It is responsible for the substrate-binding and geometric structure of the T1 copper site through the hydrogen bond network. D106 and D423 have a positive effect on TNC, which is important for basic geometry and the release of water molecules [199]. In addition, the influence of the axial bond between the metal and the amino acid residue in lacTT was explored. Compared with the wild type, replacing the axial methionine residue (Met510) with non-coordinating leucine in *E. coli* CueO laccase increased substrate accessibility at the T1 copper site, resulting in 100-fold enhanced ABTS oxidation activity [198]. Replacing the axial residues with non-coordinating ones can increase the efficiency (**k**_cat_/*K*_m_) at the T1 copper site [178]. Compared with wild-type laccase, glycine-replaced aspartic acid could increase the activity when expressed in *Pichia pastoris* [75]. In order to improve biological catalysis by laccase, Atefeh Khodakarami et al. [57] conducted site-directed mutagenesis on CotA from B. subtilis. According to their crystal structure analysis of the combination of CotA and ABTS, residues T415 and T418 near the T1 copper site were selected for mutation (T415I, T418I, T415G, T415G/T418I) in order to optimize substrate channel geometry. The results show that the substitution of glycine and isoleucine for site residues in the substrate-binding pocket can change the variant’s specificity and catalytic efficiency [198]. Three separate mutations (N298F, V290N, V290A) were introduced at or near the T1 copper site of Coelicolor A3(2) Streptomyces small laccase. Comparing the mutants with natural laccase showed that activity was closely related to the T1 copper site [198]. The three-site ligands have an effect on laccase properties. The more hydrophobic residues of the shaft ligand can increase laccase activity by 100-fold through enhanced substrate access to the T1 copper center. They can also shift the optimal pH value of laccase toward greater alkalinity (optimal pH from 6.5 to 8.0) due to the altered protonation state of His455 near the T2/T3 trinuclear cluster [159,198]. It may be that a mutation at this site affects the environment near T1 copper [166]. Some mutations cause the truncation of the protein, resulting in the deletion of domain 3 containing the T1 copper center, which affects laccase’s catalytic ability [67]. Based on sequence comparison and structural studies, Nikoo Nasoohi et al. [78] inserted three single-amino-acid substitutions (glutamate, serine, glycine) and one glycine to study the role of ASP 500 in the C-terminal laccase fragment located near the T1 copper center. The results show that substituting less sterically hindered aspartic acid for conserved residues (such as glycine) can increase the total activity of **k**_cat_ and laccase. In addition, some double variants will enhance laccase properties compared to single variants [161]. Site-directed mutagenesis was used to verify the roles of the N- and C-termini of laccase from *Pleurotus florida*; the template was selected, the fragment of the laccase gene was obtained, and site mutation was performed. The results showed that these two sites can effectively increase the catalytic efficiency and stability of laccase [189]. Zhi Li et al. [200] found that two residue positions (R178 and K433) are very important for the salt activation of catechol and dopamine activity by laccase. The specific functions of these sites facilitate the specific modification of the enzyme.

##### Hydrophobic Site

The hydrophobic environment of the T1 copper atom has an important influence on the redox potential; the stronger the former, the higher the latter [201]. With the M510L mutation in *E. coli* CueO laccase, T1 copper hydrophobicity was increased, and the redox potential was elevated from +450 mV to +520 mV [198]. The results of molecular docking studies suggest that aflatoxins (B1, B2, G1, G2) may interact with amino acid residues (His481 and Asn288) near the copper center of T1 through hydrogen bonding and hydrophobic interactions [156]. Then, the key amino acids were verified by site-directed mutagenesis. The crystal structure of corn laccase (Zmlac3) shows that there is a tight and deep substrate-binding pocket. Structural data and kinetic analyses indicate that the polarity and hydrophobicity in Zmlac3’s binding pocket have important effects on its activity [163]. The redox potential of the hydrophobic axial amino acid (Ala and Phe) mutants is higher than that of the wild type [178].

##### Glycosylation Site

Most fungal laccases are glycoproteins, with 3-10 glycosylation sites located in the conserved Asn-X-Thr/Ser [202,203]. N-glycosylation at Asn41 in Ganoderma laccase was shown to enhance thermostability by stabilizing β-sheet-rich regions through hydrogen bond networks [204]. The degree of protein glycosylation plays an important role in fungal laccase catalysis [203]. Three putative glycosylation sites (N293, N313, and N454) were used for mutation to explore their role in the specific activity of laccase Lcc9 from *Coprinopsis cinerea* in the heterologous expression of rLcc9 in *Pichia pastoris*. Asn was mutated to Gln at the three sites, and the specific activity of rlcc9 was higher than that of nlcc9. A comparison of the model structure shows that N313 is the only glycosylation site in nlcc9 [169]. An in-depth understanding of the effect of glycosylation sites on the biological characteristics of laccase is helpful for researchers seeking to modify these sites to improve certain aspects of laccase performance.

To date, studies have adopted the above-mentioned modification design method or combination strategy to modify laccases, mainly to improve their catalytic activity and substrate specificity, as well as their expression levels and stability [86,185,198,205]. On the one hand, mutants can improve the biological characteristics of laccase to obtain ideal models. On the other hand, not all mutants are forward mutants, and some may reduce laccase activity or stability, which is not conducive to production and use. For example, the M510L mutation in *E. coli* CueO enhanced laccase activity but reduced the redox potential by 80 mV [198]. Combining laccase crystal structure analysis and molecular simulation calculation technology can provide a good reference for laccase evolution. In addition, in laccase structures, site modification and changes must also be considered when establishing mutant protein stability [192].

### 4.3. Changes in Biochemical Properties of Laccase

#### 4.3.1. Laccase pH Adaptability

Laccase exhibits low stability under harsh industrial pH conditions. To address this, targeted mutagenesis near the T1 copper center and substrate-binding loops has been employed to modulate pH adaptability [86,185]. Studies related to laccase modification have shown that laccase’s optimal pH can be improved through protein or genetic engineering. Generally, compared with the wild type, the pH value of the mutant shifts towards more alkalinity [67,173,189]. Compared with the stable pH range (7.0–10.0) of the wild type, that of the variant is broader (6.0–11.0) [173]. The pH at which the enzyme is most stable increases by one unit. The pH stability of cot-A-laccases is neutral or alkaline [173], and both the wild type and the mutant have poor acid resistance. The D500G mutant retains catalytic properties similar to those of wild-type laccase and can effectively decolor synthetic dyes under alkaline conditions, displaying a higher stability at pH 9.0. For the two mutants POXA1c-R5V and POXA1cΔ13-R5V, the optimal pH values are 2.2 and 4.0, and the residual activities were 63% and 78%, respectively [189]. I. Pardo et al. [206] found that the pH of mutants with changed residues in the substrate-binding pocket, compared to that of the parent type, tends to be neutral for the substrate salicylic acid.

#### 4.3.2. Laccase Temperature Change

The Thr415, Thr418, and Gln442 sites in the mutant RB5 of strain *B. pumilus* W3 were altered through saturation mutagenesis and screening and have different effects on the biological properties of laccase. After mutating Thr415, Thr418, and Gln442, the thermal stability of some mutants remained similar to that of the wild type; only T418K improved the thermal stability of laccase. However, the thermal stability decreased with T415D [173]. Two mutants showed similar thermal stability to the wild type, and the remaining enzyme activity was about 60%. Compared with the wild-type laccase protein, the M460A mutant had a higher thermal stability (80%) [178]. At the same time, the optimal temperature of some laccase mutants did not change, but the thermal stability was reduced [157,166,178]. Laccase deglycosylation will reduce its thermal stability [169]. Among all the variants, T415I led to the highest stability. Compared with the wild-type enzyme (30 min), the half-life (t_1/2_) (60 min) of T415I was increased about 2-fold [57]. Leonardo David Herrera-Zúñiga et al. [59] proved that heat stability can be obtained through precise single-amino-acid residue mutations, and arginine mutants have better heat stability than their lysine counterparts. Glu188Tyr and Glu188Phe show significant improvements in thermal stability and ionic liquid tolerance [158]. Single-residue mutagenesis at position Tyr230 in Streptomyces coelicolor laccase (Y230R) demonstrated that replacing tyrosine with arginine broadened the range of pH tolerance (pH 3–9) while increasing ABTS oxidation activity by 104%. Structural analysis revealed that the Y230R mutation reorganized hydrogen bond networks and reduced steric hindrance in the substrate-binding pocket. Thermostability also improved due to enhanced hydrophobic packing in domain 2 [185].

#### 4.3.3. Substrate Specificity

The *K*_m_ value can reflect the substrate affinity of laccase, which is an important factor affecting the rate of substrate oxidation (Table 4); the smaller the *K*_m_ value, the greater the affinity [166]. Substrate affinity (*K*_m_), turnover rates (**k**_cat_), and catalytic efficiency (**k**_cat_/*K*_m_) are three kinetic parameters that indicate laccase’s catalytic oxidation ability [200].

Changing glycosylation sites in laccase can improve its specific activity. The enzymatic activity of deglycosylated laccase (drlcc9) is 2.63 times lower than that of rlcc9, and that of rlcc9 is about 34 times that of nlcc9 [169]. After saturation mutagenesis at Thr415, Thr418, and Gln442, two two-site mutants, T415D/Q442A and T418D/Q442A, were obtained. These obtained 3.7- and 4.43-fold increases in the decolorization rate of RB5 without a mediator, and the catalytic efficiency of T418D/Q442A on ABTS was increased 5.53 times compared to the wild type. However, the activity of the T415D/T418K mutant almost disappeared [173]. Comparing the crystal structures of the mutant and wild type revealed that the activity of the G304K mutant is about 20 times higher, which may be due to the interaction between the R-loop and the methionine-rich region [183]. When the four-residue mutants of LacTT laccase use guaiacol as the substrate, the catalytic efficiency of lacTT laccase is lower than that of the wild type [199]. Felipe de Salas et al. found [164] that 7D5 improved the catalytic activity of laccase but did not change the optimal pH or redox potential. Compared with the wild type, it has a higher **k**_cat_ (2–9-fold) and a greater affinity for DMP. Kinetic experiments with ABTS and SGZ as substrates showed that, compared with the wild type, mutants with amino acid substitutions near the copper site of T1 exhibit higher catalytic efficiency and thermal stability [57]. Compared with POXA1c, the N-terminal R5V site can increase the catalytic efficiency of laccase on ABTS and guaiacol by 2 and 3.5 times. Compared with the wild type, the double mutant POXA1cΔ13-R5V has a 4-fold increase in catalytic activity on ABTS [189]. The strain encoding the double mutant L185P/Q214K (rM4A) showed a 6-fold increase in secretase activity. Compared with rWT, the catalytic efficiency of purified rM-4A laccase on ABTS and 2,6-dimethoxyphenol is increased by 2.4 and 2.8 times, respectively [190]. LAC-V290N has a higher catalytic efficiency for 2,6-DMP (**k**_cat_/*K*_m_ = 2.226 mM^−1^s^−1^) and ABTS (**k**_cat_/*K*_m_ = 1.874 mM^−1^s^−1^) compared to wild-type SLAC (**k**_cat_/*K*_m_ = 1.615 mM^−1^s^−1^ for 2,6-DMP and **k**_cat_/*K*_m_ = 1.611 mM^−1^s^−1^ for ABTS) [75].

The increase in laccase activity in the co-culture system compared to the control group may be due to the up-regulation of the three laccase genes of *P. eryngii* var. ferulae, namely *lacc1* (Cluster-1041.14150), *lacc2* (Cluster-1041.48984), and *lacc12* (Cluster-1041.55007) [73]. Compared to the group without farnesol, the laccase activity after treatment increased by 1.92 times [207]. The laccase enzyme activity of the strongest chemically mutagenized mutant strain increased by 3 times [197]. At present, studies mainly focus on analyzing the influence of related structures near T1 copper in laccase. Based on these research foundations, combined with laccase structure–function analysis, specific mutagenesis can be performed on target laccases with specific characteristics to obtain results with optimal biological functions.

## 5. Conclusions

As pivotal members of the multicopper oxidase family with broad-spectrum substrate catalytic capacity (phenolic compounds, aromatic amines, lignin derivatives) and green catalytic properties (water as the sole by-product), microbial laccases demonstrate the potential to revolutionize bioremediation, food processing, and green manufacturing. Given the limitations of native laccases, including their poor thermal/pH stability, inefficient heterologous expression, and high-scale production costs, this study systematically evaluates the strengths and weaknesses of fungal (*Pichia pastoris*, *Trichoderma reesei*) and bacterial (*Escherichia coli*) expression systems and proposes targeted optimization strategies. Furthermore, it synthesizes the technical foundations for protein-engineered laccase modifications, elucidating the structural basis and active-site engineering principles. These insights provide actionable directions for accelerating the development and commercialization of microbial laccases, paving the way for sustainable and eco-friendly biotechnological solutions.

## 6. Prospects

Due to the successful heterologous expression and modification of laccase, this enzyme has been extensively applied in various fields. In the food industry, laccase has been used to clarify juice [6,208] and improve bread quality [209,210,211,212], and it has shown promise as a sustainable option for engineering biodegradable and functional food packaging materials [213,214,215]. For bioremediation in a polluted environment, laccase has been applied for the degradation of different pollutants, such as 2-Methylisoborneol [216], trichloroethylene [217], and tetracycline [218,219,220]. In paper pulping, laccase was used for pulp bleaching [221,222,223], where it improved the whiteness, tensile index, and breaking length of the paper. In addition, laccase can be employed in the fabrication of biosensors for the detection of catechol [224] and Bisphenol A [225].

This study elucidates the heterologous expression characteristics of laccases in bacterial and fungal expression systems. The expression efficiency of laccases is intrinsically linked to the properties of host systems. Bacteria-derived laccases fully exploit the advantages of rapid proliferation and high-yield expression in bacterial systems, demonstrating strong competitiveness for large-scale production. However, current bacterial induction systems heavily rely on isopropyl β-D-1-thiogalactopyranoside (IPTG), the prohibitive costs of which constrain industrial applications. Developing high-efficiency, low-cost bacterial inducers is a critical future research direction. While *Escherichia coli* remains the predominant bacterial expression host, its biosafety risks limit its applications in sensitive fields, such as food processing. Consequently, engineering non-pathogenic hosts has emerged as a strategic optimization pathway for specialized industrial scenarios.

In fungal expression systems, *Pichia pastoris* is widely recognized as an excellent host for recombinant protein production. Although methanol-induced systems enable high-efficiency laccase secretion, the stringent requirements for aerobic fermentation and the toxicity associated with methanol necessitate urgent solutions. Exploratory efforts to develop non-methanol induction systems or constitutive expression systems, combined with the engineering of safer filamentous fungal hosts, demonstrate the potential to overcome these limitations.

To advance the molecular engineering of laccase, it is imperative to establish a comprehensive structure–function database that integrates the genetic sequences and biochemical characterization data of critical domains. Building upon this foundation, AI-driven rational design models, such as deep learning frameworks for predicting enzyme–host compatibility and catalytic hotspots, could be developed to enable intelligent recommendation systems. These systems would guide the sequential workflow, encompassing source strain selection, regulatory target identification, and modification strategy implementation, thereby accelerating the development of high-performance laccase variants.

## Figures and Tables

**Table 1:** Bacterial heterologous expression system.

Host	Laccase Source	Expression Strategy	Vector	Inducer	Reaction Substrate	Activity (U/L, ABTS)	Ref.
*E. cloni 10G*	*Geobacillus* sp.	Extracellular	pRham N-His SUMO Kan	--	ABTS	37	[104]
*E. coli Top10*	*Sordaria macrospora*	Extracellular	pET-30a-LacSM	Cu^2+^	SGZ, ABTS, 2,6-DMP, and guaiacol	239	[105]
*Escherichia coli BL21 (DE3)*	*Y. enterocolitica*	Intracellular	pTZ57R/T	IPTG (1 m M)	ABTS	3671	[106]
*Escherichia coli BL21 (DE3)*	*Geobacillus* sp.	Intracellular	Topo blunt vector	IPTG (0.3 mM)	SGZ, ABTS, 2,6-DMP and guaiacol	--	[22]
*E. coli Rosetta (DE3)*	*Pseudomonas Species*	Extracellular	pRSETB	IPTG (1 m M)	ABTS and guaiacol	--	[107]
*Escherichia coli BL21*	*Pleurotus ostreatus*	Extracellular	Pet-22b (+)	IPTG (1 m M) and CuSO_4_ (0.25 mM)	ABTS	1539	[108]
*E. coli BL21(DE3)*	--	Extracellular /Intracellular	pET28a	IPTG (0.6 mM) and CuCl_2_ (0.25 mM)	ABTS	--	[109]
*E. coli M15 (pREP4)*	*Streptomyces puniceus*	Extracellular	pQE-30	IPTG	--	--	[103]
*E. coli BL21(DE3)*	*Bacillus amyloliquefaciens*	Extracellular /Intracellular	pET-20 (b) + /lac and pPICZαB/lac	IPTG (0.03 mmol/L)	ABTS and SDZ	20255	[99]
*E. coli M15 (pREP4)*	*Catenuloplanes japonicus*	Intracellular	pQE-30	IPTG (0.2 mM) and CuSO_4_ (1 mM)	ABTS and 2,6-DMP	--	[48]
*E. coli DH5α*	seven bacteria laccase genes	Extracellular	pET-Ompa and pET-Lpp	IPTG (1 mM)	SDZ, SMZ and SMX	--	[110]
*Escherichiacoli DH10B*	*Streptomyces viridochromogenes*	Intracellular	pAL-TA	IPTG (0.1 mM) and CuSO_4_ (0.25 mM)	ABTS	--	[101]
*E. coli BL21 (DE3)*	*Bacillus vallismortis*	Extracellular	pET-23a	Cu^2+^ (0.25 mM)	ABTS	1580	[98]
*E. coli DH5α*	*Geothermobacter hydrogeniphilus*	Intracellular	pET-22b	IPTG (0.2 mM)	--	--	[111]
*E. coli DH5α and E. coli BL21 (DE3)*	*Bacillus mojavensis*	Intracellular	pET-14b	IPTG (0.3 mM)	ABTS, 2,6-DMP and SDZ	--	[112]
*Streptomyces lividans and Bacillus subtilis*	*Streptomyces coelicolor* *, Streptomyces viridosporus and Amycolatopsis*	Intracellular	pBE-S	CuSO_4_ (100 µM)	ABTS	1950	[113]
*E. coli BL21 (DE3)*	*Bacillus vallismortis*	Extracellular /Intracellular	pET-28a	Methanol (6%, *v*/*v*)	ABTS	1545.6	[114]
*E. coli BL21 (DE3)*	*Bacillus cereus and Ochrobactrum pseudintermedium*	Intracellular	pET28a (+)	IPTG (0.2 mM)	ABTS	7.54	[115]
*E. coli BL21 (DE3)*	*Ochrobactrum* sp.	Intracellular	pUC59 and pET22b (+)	IPTG (0.4-1 mM)	ABTS, 2,6-DMP and SDZ	--	[116]

**Table 2:** Fungal heterologous expression system.

Host	Laccase Source	Expression Strategy	Vector	Inducer	Reaction Substrate	Activity (U/L, ABTS)	Ref.
*Pichia pastoris*	*Cerrena* sp.	Extracellular	pMD18-T	Cu^2+^ (0.25 mM)	ABTS	--	[133]
*Pichia pastoris*	*Aspergillus* sp.	Extracellular	pPIC9 K-Lac, pPIC9 K-MnP and pPIC9 K-LiP	--	ABTS, and veratryl alcohol	--	[134]
*Pichia pastoris*	*Madurella mycetomatis*	Extracellular	pPICZA and pPICZαA	Methanol (1%)	ABTS, SGZ and 2,6-DMP	--	[7]
*Pichia pastoris*	*Laccaria bicolor*	Extracellular	pMD18-T	--	ABTS	--	[135]
*Pichia pastoris*	*Coprinopsis cinerea*	Extracellular	pPIC9K	Methanol	ABTS	3138	[118]
*Pichia pastoris*	*Grifola frondosa*	Extracellular	pPICZA	CuSO_4_	ABTS and 2,6-DMP	--	[136]
*Pichia pastoris*	*Phlebia brevispora*	Extracellular	pGEM-T Easy	Methanol (0.5%)	ABTS	--	[127]
*Pichia pastoris*	*Pleurotus ostreatus*	Extracellular	pPIC3.5K	Methanol	ABTS	500	[137]
*Pichia pastoris*	*Rigidoporus* sp.	Extracellular	--	CuSO_4_ (0.3 M)	ABTS	--	[138]
*Saccharomyces cerevisiae*	*Trametes versicolor*	Extracellular	pYES2	Cu^2+^	ABTS	45	[130]
*Saccharomyces cerevisiae*	*Aspergillus niger*	Extracellular	--	--	ABTS	--	[138]
*Saccharomyces cerevisiae*	*Agrocybe pediades*	Extracellular	pJMP9.1	CuSO_4_ (2 mM)	ABTS	778	[139]
*A. nidulans*	*Pycnoporus sanguineus*	Extracellular	pMD18-t	Cu^2+^ (0.1 mmol/L)	--	--	[140]
*Trichoderma atroviride*	*Trametes(Pycnoporus)* *sanguineus*	Extracellular	pGEM-T Easy	CuSO_4_ (100 μM)	ABTS, guaiacol, syringaldazine and o-dianisidine	--	[120]
*Trichoderma reesei*	*Pycnoporus sanguineus*	Extracellular	pD915	Lactose (2% *w*/*v*)	ABTS	--	[132]
*Pichia pastoris*	*Fusarium oxysporum*	Extracellular	pPIC9K	Cu^2+^ (0.08 Mm)	ABTS	21966	[141]
*Pichia pastoris*	*Trametes cinnabarina*	Extracellular	--	--	ABTS	2851	[142]
*Pichia pastoris*	*Trametes hirsuta*	Extracellular	--	Methanol	ABTS	2590	[143]
*Pichia pastoris*	*Coprius cinerea*	Extracellular	pPICZB	--	ABTS	2760	[144]
*Pichia pastoris*	*Pleurotus ostreatus*	Extracellular	Vector		ABTS	285.7	[145]
*Komagataella phaffii*	*Trametes versicolor*	Extracellular	pMD18-T	CuSO_4_ (0.1 mM) and Methanol	ABTS	--	[146]

**Table 3:** Comparison of laccase expression in bacteria and fungi.

Host	Culture Cycle (h)	Range of Enzyme Activity (U/L, ABTS)	Sources of Laccase Genes	Expression Strategies
Fungi	24–48	200–3000	Bacteria and fungi	Primarily extracellular
Bacteria	16–36	200–2000	Bacteria and fungi (primarily bacteria)	Intracellular and extracellular expression

**Table 4:** Influence of mutation sites on laccase substrate specificity.

Source Strain	Mutant Protein	Control	Mutant Position	WT *K*_m_	*K*_m_	WT Specific Activity	Mutant k_cat_/*K*_m_	Substrate	Ref.
*Escherichia coli*	R178V, K433T	Lac15	R178 and K433	2031.22, 125.51	1736.25, 182.98				[200]
*Thermus thermophilus*	M460L	lacTT	Axial residue	378.49	72.40	15.85	3.69	guaiacol	[178]
*Streptomyces coelicolor* A3 (2)	SLAC-V290N	SLAC	T1 copper site	5.088	1.999	1.615	2.226	2,6-DMP	[166]
*Pleurotus ostreatus*	POXA1cΔ13-R5V	Wild-type	N- and C-terminals	0.97	1.13	2.67	25.98	Guaiacol	[189]
*Pichia pastoris*	D500G	04lac	-	44.0	58.2	-	-	ABTS	[75]
*Bacillus* HR03	T415G, T415I, T418I, T415G/T418I	Native	T415 and T418	6.7	1.1, 4.1, 3.4, 2.3	2.34	9.54, 0.81, 0.13, 0.15	SGZ	[57]
*Fusarium oxysporum*	4C1, 4A9	Gr2	-	739.0	504.4, 271.7	0.02	0.03, 0.22	DMP	[72]
*Coprinopsis cinerea*	N313Q/N454Q	nLcc9	N-glycosylation sites	1.10 × 10^−5^	1.96 × 10^−5^	1.95 × 10^7^	3.93 × 10^7^	ABTS	[169]
*basidiomycetes*	C14F12, CA32F1	3A4	Residues at substrate-binding pocket (six in total)	7.0	14.2, 9.9	22	17.7, 26	SA	[206]

WT: wild type.

## Data Availability

No new data were created or analyzed in this study. Data sharing is not applicable to this article.
